# A Web-Based Sexual Violence Bystander Intervention for Male College Students: Randomized Controlled Trial

**DOI:** 10.2196/jmir.3426

**Published:** 2014-09-05

**Authors:** Laura F Salazar, Alana Vivolo-Kantor, James Hardin, Alan Berkowitz

**Affiliations:** ^1^Georgia State UniversitySchool of Public HealthAtlanta, GAUnited States; ^2^Centers for Disease Control and PreventionDivision of Violence PreventionAtlanta, GAUnited States; ^3^University of South CarolinaDepartment of Epidemiology and BiostatisticsColumbia, SCUnited States; ^4^Independent ConsultantMount Shasta, CAUnited States

**Keywords:** Internet, sex offenses, rape, universities, students, public health

## Abstract

**Background:**

Bystander intervention approaches offer promise for reducing rates of sexual violence on college campuses. Most interventions are in-person small-group formats, which limit their reach and reduce their overall public health impact.

**Objective:**

This study evaluated the efficacy of RealConsent, a Web-based bystander approach to sexual violence prevention, in enhancing prosocial intervening behaviors and preventing sexual violence perpetration.

**Methods:**

A random probability sample of 743 male undergraduate students (aged 18 to 24 years) attending a large, urban university located in the southeastern United States was recruited online and randomized to either RealConsent (n=376) or a Web-based general health promotion program (n=367). Participants were surveyed online at baseline, postintervention, and 6-months postintervention. RealConsent was delivered via a password-protected Web portal that contained six 30-minute media-based and interactive modules covering knowledge of informed consent, communication skills regarding sex, the role of alcohol and male socialization in sexual violence, empathy for rape victims, and bystander education. Primary outcomes were self-reported prosocial intervening behaviors and sexual violence perpetration. Secondary outcomes were theoretical mediators (eg, knowledge, attitudes).

**Results:**

At 6-month follow-up RealConsent participants intervened more often (*P*=.04) and engaged in less sexual violence perpetration (*P*=.04) compared to controls. In addition, RealConsent participants reported greater legal knowledge of sexual assault (*P*<.001), greater knowledge of effective consent (*P*<.001), less rape myths (*P*<.001), greater empathy for rape victims (*P*<.001), less negative date rape attitudes (*P*<.001), less hostility toward women (*P*=.01), greater intentions to intervene (*P*=.04), less hyper-gender ideology (*P*<.001), less positive outcome expectancies for nonconsensual sex (*P*=.03), more positive outcome expectancies for intervening (*P*<.001), and less comfort with other men’s inappropriate behaviors (*P*<.001).

**Conclusions:**

Our results support the efficacy of RealConsent. Due to its Web-based format, RealConsent has potential for broad-based dissemination thereby increasing its overall public health impact on sexual violence.

**Trial Registration:**

Clinicaltrials.gov: NCT01903876; http://clinicaltrials.gov/show/NCT01903876 (Archived by WebCite at http://www.webcitation.org/6S1PXxWKt).

##  Introduction

Sexual violence is a major public health problem with long-term mental, physical, and social effects for victims [1-3]. Sexual violence encompasses a breadth of personal violations ranging from minor, nonconsensual noncontact acts, such as exhibitionism or verbal sexual harassment to sexual coercion, up to severe acts, such as attempted or completed nonconsensual oral, genital, or anal penetration [4,5]. The 2010 National Intimate Partner and Sexual Violence Survey found that 18.3% of women in the United States have been raped in their lifetime [1]. Younger age has been shown to be a significant factor in sexual violence risk. Recent research has shown that 80% of female victims of sexual violence reported that their first rape occurred before the age of 25 years [1]. According to results from the National College Women Sexual Victimization study, 2.8% of female college students surveyed reported either a completed rape or attempted rape in the previous 6 months, which equates with an incidence rate of 5.6% per year [6]. Research has also shown that the majority of sexual victimizations occur by perpetrators the victim knows [6-8].

To combat the problem of sexual violence, most prevention and intervention programs have focused on college populations and have shifted efforts recently to target elements in the environment rather than solely targeting individual characteristics of perpetrators or victims. The bystander model is one such approach. It is a theoretical model of community-level change that targets community members to intervene actively in situations that may be harmful and engages them to be accountable and to take action [9-11]. In fact, bystander intervention programs applied to dating and/or sexual violence interventions have proliferated in the past 5 years [12-20]. Most of these have involved a small-group format (eg, workshops), whereas a few involved a localized social marketing campaign. Subsequent evaluations demonstrated that the bystander model, in some cases, is effective in promoting active bystander behaviors and in changing social norms [13].

As promising as these interventions are, because of their small-group in-person format, they are resource-intensive and limited in their reach and sustainability. Alternatively, the use of the Internet as an effective medium to deliver health-related interventions has emerged [21,22] and offers significant advantages over in-person interventions, such as lower cost of intervention delivery, greater reach, maintenance of fidelity, the possibility of delivery in a wide range of settings, and ability to tailor content to a variety of users [23-26]. There are Web-based treatment programs designed to ameliorate depression [27,28], obesity [29,30], eating disorders [31], alcohol abuse [32-36], smoking [37], sexual risk reduction [38], and posttraumatic stress disorder [39]. In a study of college students, results showed that a Web-based format was preferable over a practitioner-delivered intervention for a hazardous drinking prevention program [33].

To date, there have been no Web-based sexual violence prevention programs that target male college students and incorporate the bystander approach, which have also been tested using a true experimental design and with sexual violence as an outcome. In response, RealConsent, a Web-based sexual violence prevention program incorporating a bystander approach, was developed and tested for its efficacy. This paper reports primary and secondary outcomes from a randomized controlled trial (RCT) of RealConsent. It was hypothesized that participants randomized to receive the RealConsent program would report greater increases in self-reported prosocial intervening behaviors and fewer incidents of sexual violence perpetration in comparison to participants randomized to receive an attention-placebo comparison program.

## Methods

### Recruitment and Study Design

An RCT was implemented at a Georgia State University, a large, urban university located in Atlanta, GA. Study procedures were approved by the participating sites’ Institutional Review Boards. Participants provided informed consent; however, documentation was waived because of online recruitment.

Eligible participants were male undergraduates aged 18 to 24 years, single, who self-reported being either heterosexual or bisexual; exclusion criteria were graduate student status and homosexual sexual orientation. Active recruitment began February 2010 and ended in April 2010, and was accomplished through email messages from the principal investigator’s university email address sent to randomly selected students. The sampling frame to generate the random sample was a list of student names obtained from the university’s Office of Legal Affairs. The list included only first and last names, email addresses, and year of birth for all undergraduate students enrolled during 2009-2010 school year (N=21,280). To pare down the sampling frame, the list was sorted by birth year and all students born on or before January 1, 1984 were deleted from the list. Next, the list was sorted alphabetically by first name and an online resource (Baby Name Guesser) was used to determine likelihood of a name belonging to a male. This process resulted in a final sampling frame of 8458 male students. A random sample function from SPSS version 18.0 was used to select groups for invitation to participate in the RCT. Over the course of 10 weeks, 5 groups of randomly selected email addresses ranging in size from 300-3000 were emailed an invitation to participate.

Email invitations included a link to a website that included a short description of the study that blinded participants to the research questions and a short screener to determine eligibility. Eligible students were directed to a webpage that contained the informed consent form. Students were told that the purpose of the study was to “test multimedia, Web-based interactive programs designed for male college students.” They were also told that the study would be anonymous (ie, email addresses could not be linked to their user ID or to their responses on the survey). Participants who provided consent were then directed to the RealConsent Web portal to register and obtain a username and set their password. After registering, simple randomization was implemented using a customized algorithm that assigned participants to either the experimental condition (RealConsent) or to an attention-placebo comparison condition. Participants were then directed to complete the baseline online survey. Following completion of the survey, they were directed to either the RealConsent program or to the comparison program where they could begin the modules immediately or at a later time. Participants were asked to complete a survey postintervention and at 6-month follow-up. Weekly email reminders were sent to participants reminding them to complete each survey. The Web portal also allowed participants to send emails to a project coordinator and post messages if they needed assistance or had questions. Participants were paid US $25 via PayPal for completing each online survey.

### Interventions

#### Experimental Intervention

The content for RealConsent was based on several complementary theoretical frameworks (social cognitive theory [40], social norms theory [41], and the bystander educational model [42]) as well as extensive formative research with the targeted population. RealConsent had two primary goals: (1) to increase prosocial intervening behaviors that reduce risk for sexual violence perpetration (eg, expressing disapproval when a peer is verbally disparaging toward women, attempting to stop a peer who tries to be coercive/violent) and (2) to prevent sexually violent behaviors toward women. These goals were to be achieved by affecting theoretically and empirically derived mediators, such as increasing knowledge of and skills for safely intervening, correcting misperceptions in normative beliefs, affecting negative attitudes toward date rape, increasing knowledge of the elements constituting effective consent for sex, affecting masculine gender roles, enhancing communication skills, and increasing empathy for victims of sexual assault.

**Figure 1:**
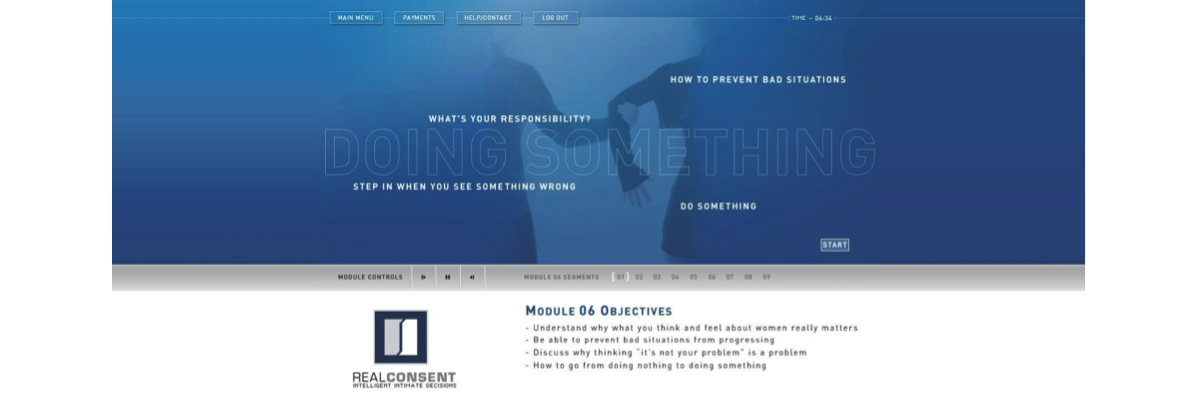
Screenshot of RealConsent’s bystander intervention module.

RealConsent was delivered via a password-protected Web portal (see Figure 1 for screenshot) and consisted of six 30-minute modules with each module ranging in number of segments (1-14) and types of activities with diverse actors and appropriate language. Each of the modules involved interactivity, didactic activities, and episodes of a serial drama, which allowed us to model positive behaviors and illustrate both positive and negative outcome expectations for intervening and for perpetrating violence against women. Behaviors modeled in the serial drama included intervening, communicating with female sex partners, and obtaining effective consent for sex.

RealConsent was also programmed so that participants could not skip or click-through segments within each module. Participants were allowed to complete the modules at their own pace, but were encouraged via email to complete all modules within 3 weeks. Following the completion of each module, participants were immediately assessed and compensated US $10 for providing their acceptability and feedback on the module. These procedures ensured that each participant at follow-up had completed each module in its entirety.

#### Attention-Placebo Comparison Intervention

The comparison condition involved a Web-based, general health promotion program titled Health Connection developed by the ISA Group [43]. The Health Connection program is an online, multimedia, health promotion program with 4 primary modules: stress management, fitness, weight management/nutrition, and substance abuse. Each program module is approximately 45 minutes long and audio narrated and approximated RealConsent in intensity, format, and time duration.

### Measures

#### Overview

Primary outcome measures included prosocial intervening behaviors and sexual violence perpetration; secondary outcome measures were a host of theoretical mediating variables linked to the intervention activities. The primary behavioral outcomes, legal knowledge of sexual assault/rape and knowledge of effective consent for sex, are considered indexes and comprise items deemed “causal indicators,” which indicates items are not necessarily correlated; thus, internal reliability was not calculated for these indexes because it is not an appropriate method to assess reliability [44,45]. The remaining theoretical mediators, considered “scales” and comprised of items deemed “effect indicators,” should theoretically be highly intercorrelated. Cronbach alpha was calculated for the scale measures [44].

#### Primary Outcome Measures

Prosocial intervening behaviors were assessed with the Reactions to Offensive Language and Behavior (ROLB) index that measures whether or not men confronted inappropriate behaviors of other men [46,47]. We used the 7-item self-behavior subscale plus an additional 8 items [9], which directly reflected the content of RealConsent. A series of 15 potential intervening situations were presented and participants were asked to indicate whether they had experienced this situation (yes/no), whether they had “ever” intervened (at baseline), or whether they had intervened “within the past 6 months” (at 6-month follow-up). For each participant, a percent score was calculated that represented the total number of situations in which they intervened out of the total number of situations encountered multiplied by 100. For participants who indicated they had not encountered any of the 15 situations, their data were dropped from analyses.

Sexual violence was assessed with the sexual coercion subscale from the Revised Conflict Tactics Scale (CTS2) [48]. The CTS2 sexual coercion subscale is a 7-item questionnaire that assesses a range of sexually coercive behaviors in which participants are asked to indicate whether they had engaged in a sexually abusive tactic (eg, “I used force like hitting, holding down, or using a weapon to make a woman have sex”) within a certain timeframe. We used “ever” at baseline and “within the past 6 months” at the 6-month follow-up.

#### Secondary Outcome Measures

The secondary outcome measures included legal knowledge of assault/rape [49], knowledge of effective consent for sex, self-efficacy to intervene [9,46], intentions to intervene, outcome expectancies for intervening behaviors [9], normative beliefs regarding sexual violence toward women [46,47], rape myths [50], gender-role ideology [51], empathy for rape victims [52], hostility toward women [53], attitudes toward date rape [54], and outcome expectancies for engaging in nonconsensual sex. Table 1 provides additional information about these secondary measures, including the mean and standard deviation, Cronbach alpha, the number of items, response options, and a sample item.

**Table 1:** Description of secondary outcomes/psychosocial mediators and means (SD) at baseline.

Psychosocial mediators	Mean (SD)	Range	Cronbach alpha	Items, n	Response options	Sample item
Legal knowledge assault/rape^a,b^	4.57 (1.75)	0-9	—	9	True/false	“In the state of Georgia, it is always legal to engage in sexual intercourse with a person under the age of 16 so long as he or she gives consent?”
Knowledge effective consent for sex^a,b^	11.58 (2.46)	0-14	—	14	True/false	“If a woman doesn’t physically resist sex, she has given consent.”
Self-efficacy to intervene	88.32 (22.27)	18-126	.95	18	1 (not at all confident) to 7 (extremely confident)	“Indicate my displeasure when I hear a sexist comment.”
Intentions to intervene	52.39 (12.31)	15-75	.94	15	1 (not at all likely) to 5 (extremely likely)	“If I saw a man being verbally abusive toward a woman, I would intervene.”
Outcome expectancies intervening	28.49 (4.60)	8-40	.80	8	1 (strongly disagree) to 5 (strongly agree)	“If I intervene, I can prevent someone from being hurt.”
Self-comfort with men’s inappropriate behaviors (normative beliefs)	33.11 (10.77)	8-56	.89	8	1 (not at all comfortable) to 7 (extremely comfortable)	Estimate how comfortable you feel with each of the following situations...”You are getting ready to go on a date when your roommate walks in with a bottle of tequila. He says to you, ‘if you give her a couple shots of this, she’ll loosen up.’”
Rape myth acceptance	36.28 (10.36)	17-85	.86	17	1 (not at all agree) to 5 (very much agree)	“Rape happens when a man’s sex drive gets out of control.”
Outcome expectancies engaging in rape	58.38 (10.96)	14-70	.87	14	1 (strongly disagree) to 5 (strongly agree)	If I engage in sex without clear consent...”I would feel more like a man.”
Empathy for rape victims	68.87 (9.72)	19-95	.78	19	1 (strongly disagree) to 5 (strongly agree)	“In general, I feel that rape is an act that is not provoked by the rape victim.”
Hostility toward women	3.66 (2.49)	0-10	.73	10	True/false	“I feel that many times women flirt with men just to tease them or hurt them.”
Date rape attitudes	128.65 (24.95)	50-250	.93	50	1 (strongly disagree) to 5 (strongly agree)	“Most women don’t understand that sexual jokes and innuendos are only for fun and are harmless.”
Hyper-gender ideology	46.49 (11.28)	19-95	.89	19	1 (strongly disagree) to 5 (strongly agree)	“If men pay for a date, they deserve something in return.”

^a^ Mean represents the mean percent correct.

^b^ Cronbach alpha not calculated for this index measure.

### Data Analysis

Sample size calculations for the primary behavioral outcomes were estimated to guarantee that power would be at least .75 for the detection of small effect sizes [55]. Under a 2-group design and assuming 10% attrition over the 6-month follow-up period required enrolling at least 340 participants in each study condition to detect the specified effect size with power of .83.

Analyses were performed only on prespecified hypotheses using an intention-to-treat protocol in which participants were analyzed according to their original assigned study conditions [56,57]. At baseline, descriptive statistics were calculated to summarize sociodemographic variables, theoretical mediators, intervening, and sexual perpetration behaviors between study conditions. Differences between study conditions were assessed using *t* tests for continuous variables and chi-square analyses for categorical variables [58]. Variables in which differences between study conditions approached statistical significance (*P*<.10) or that were theoretically or empirically identified as potential confounders were included as covariates in the models. The effectiveness of RealConsent on primary outcomes was analyzed for the 6-month period examining changes in behavioral outcomes across 2 time points (baseline and 6 months); the postintervention time point was not examined because we did not expect behavioral changes immediately following completion of RealConsent. The effects analysis for primary outcomes used logistic regression to compute adjusted odd ratios (AORs) for dichotomous outcomes [59] and analyses of covariance (ANCOVA) [60] to compute adjusted means and mean differences for continuous outcomes. Each of these approaches included the corresponding baseline measure for the specific outcome as a covariate in the analysis. Effect sizes with Cohen’s *d* were also calculated using Practical Meta-Analysis Effect Size Calculator [61].

To assess effects of RealConsent on secondary outcomes, linear generalized estimating equation (GEE) regression models were estimated to control for repeated within-subject measurements and examined changes in outcomes across 3 time points (baseline, postintervention, and 6-months); these models assuming exchangeable correlation are equivalent to random-effects models which include a second variance component for participants. These models admitted a differential number of observations on study participants over the longitudinal course of observation and included a time-independent variable (treatment condition) as well as time-dependent variables (covariates and outcomes). The models adjusted for the corresponding baseline measure for each outcome and other covariates to obtain adjusted mean differences used to assess the effect of the intervention on each continuous outcome. Additionally, an indicator for the time period was included in the model to capture any unaccounted temporal effects [62,63]. The 95% confidence intervals (CIs) around the adjusted mean differences and the corresponding *P* values were also computed. To obtain adjusted means and mean differences, models were repeatedly estimated from the bootstrap samples in which samples were drawn with replacement at the level of the participant. For each model, adjusted means were calculated and standard errors were then calculated from the collection of bootstrap results [64]. Percentage of relative change for continuous variables was computed as the difference between the adjusted means for each condition divided by the adjusted mean for the comparison condition. Analyses were performed using Stata statistical software, version 13 (StataCorp LP, College Station, TX, USA), and SAS version 9.3 (SAS Institute Inc, Cary, NC, USA).

## Results

### Recruitment

**Figure 2:**
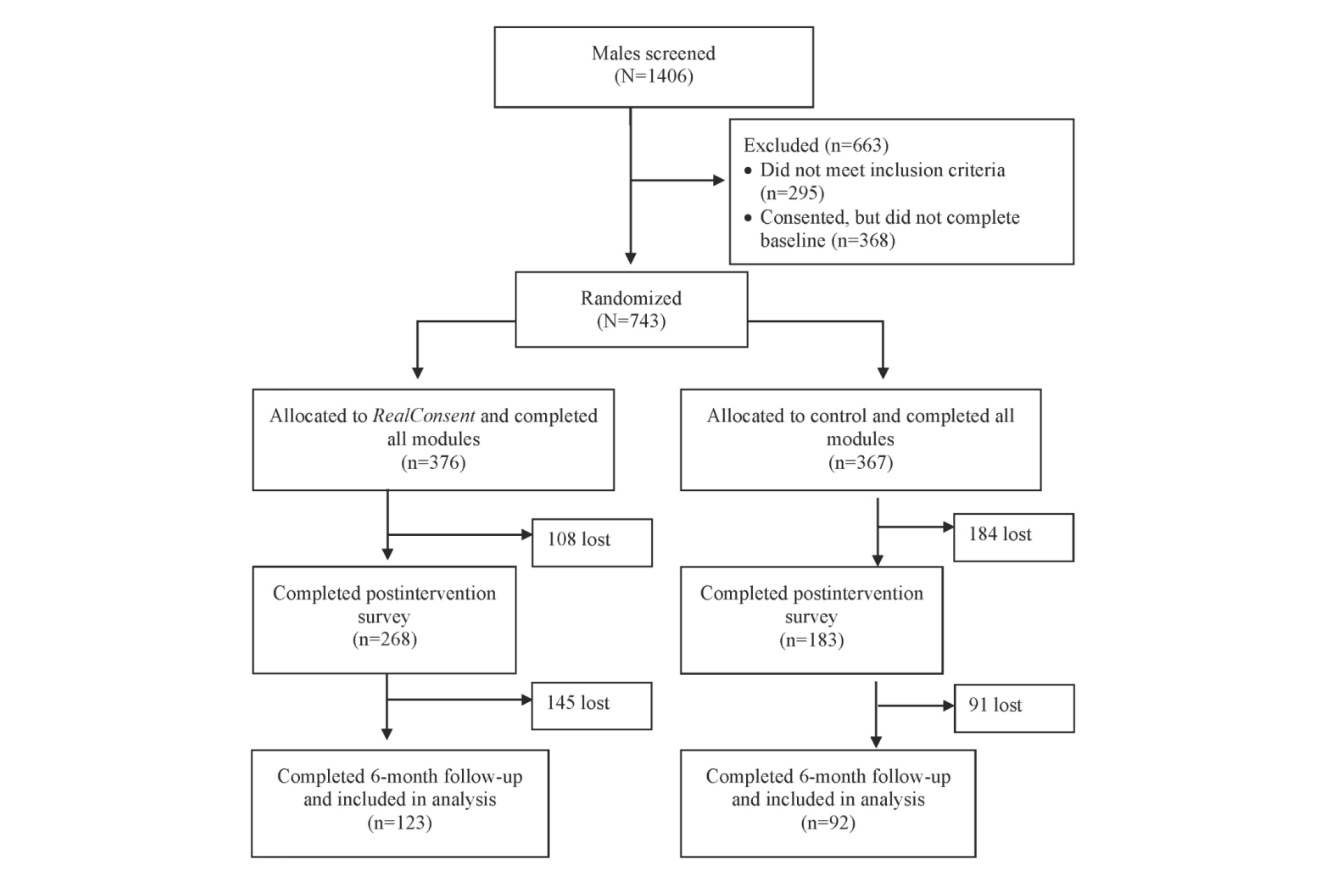
CONSORT diagram.

The recruitment process resulted in 1406 male college students who were screened. Of those, 295 (20.98%) were not eligible and 1111 (79.02%) accepted and consented. There was some attrition (33.12%, 368/1111) between initially agreeing and subsequently completing the baseline survey. The final sample resulted in 743 eligible students who were randomized and completed baseline. At postintervention, 451 of 743 (60.7%) completed the follow-up survey. Attrition at this time point was loss to follow-up. At 6-month follow-up, 215 of 743 (28.9%) completed the survey and were included in analyses (Figure 2). Attrition at this final time point was because of loss to follow-up, but also because the trial ended prematurely. More information is provided in the Discussion section.

### Participant Characteristics

#### Overview

The average age was 20.38 years (SD 1.67). Racial breakdown of participants was white (44.1%, 328/743), African American (22.3%, 166/743), Asian American (19.6%, 146/743), and Hispanic (10.8%, 80/743). Most participants were full-time students (92.1%, 684/743) and single (75.2%, 559/743); a small number were members of fraternities (12.1%, 90/743) or athletic teams (8.5%, 63/743). Overall, the prevalence of sexual violence perpetration at baseline was 32.2% (234/727). The mean percent prosocial intervening behaviors at baseline was 72% (SD 25%), meaning male participants self-reported that they intervened on average 72% of the time when exposed to a situation.

Table 2 provides data on the breakdown by sociodemographic variables, proposed mediators, and outcome behaviors for both conditions. Participants in the comparison condition reported higher levels of hostility toward women (mean 3.89, SD 2.54 vs mean 3.42, SD 2.43; *P*=.01), higher average sexual coercion tactics used (mean 0.76, SD 1.29 vs mean 0.53, SD 1.07; *P*=.01), and were more likely to ever report sexual coercion perpetration (37.0%, 134/362 vs 27.4%, 100/365; *P*=.006) than RealConsent participants. These variables at baseline were controlled for in subsequent analyses.

**Table 2:** Baseline equivalence of RealConsent and attention-placebo comparison condition participants (N=743).

Characteristics		RealConsent intervention (n=376)	Attention-placebo comparison (n=367)
**Sociodemographics**			
	Age (years), mean (SD)	20.42 (1.69)	20.33 (1.66)
	International student,^a^ n (%)	9 (2.4)	18 (4.9)
	Member of a fraternity,^a^ n (%)	37 (9.9)	53 (14.6)
	Member of an athletic team at college, n (%)	29 (7.8)	34 (9.4)
**Race, n (%)**			
	White	170 (45.2)	158 (43.1)
	African American or black	83 (22.1)	83 (22.6)
	Asian or Pacific Islander	73 (19.4)	73 (19.9)
	Hispanic or Latino	38 (10.1)	42 (11.4)
	American Indian, Alaskan native, or native Hawaiian	12 (3.2)	11 (3.0)
Frequency of vaginal intercourse, mean (SD)^a^		7.55 (12.75)	8.38 (13.03)
**Grade point average,** ^a^ **n (%)**			
	<2.0	8 (2.1)	14 (3.8)
	2.0-2.9	112 (29.8)	124 (33.8)
	3.0-3.4	163 (43.4)	150 (40.9)
	3.5-4.0	93 (24.7)	79 (21.5)
**Residence status,** ^a^ **n (%)**			
	Campus or residence hall	80 (21.5)	80 (22.0)
	Fraternity house	2 (0.5)	6 (1.6)
	Other university housing	0 (0.0)	4 (1.1)
	Off-campus housing such as own apartment	141 (37.9)	123 (33.8)
	Parent’s or guardian’s home	144 (38.7)	149 (40.9)
	Other	5 (1.3)	2 (0.5)
**Relationship status,** ^a^ **n (%)**			
	Single	289 (77.7)	270 (74.2)
	Married or has domestic partner	1 (0.3)	6 (1.6)
	Engaged or in committed relationship	82 (22.0)	87 (23.9)
	Separated	0 (0.0)	0 (0.0)
	Divorced	0 (0.0)	1 (0.3)
**Primary behavioral outcomes**			
	Sexual coercion perpetration, mean (SD)^a^	0.53 (1.07)	0.76 (1.29)
	Sexual coercion perpetration, dichotomized^a^ n(%)	100 (27.4)	134 (37.0)
	Prosocial intervening, mean percent (SD)	72 (25)	72 (24)
**Secondary outcomes (mediators), mean (SD)**			
	Legal knowledge of assault/rape	4.54 (1.73)	4.61 (1.78)
	Knowledge of effective consent for sex	11.69 (2.44)	11.46 (2.49)
	Self-efficacy to intervene	88.74 (20.93)	87.88 (23.60)
	Intentions to intervene	52.68 (11.85)	52.09 (12.78)
	Outcome expectancies for intervening	28.72 (4.33)	28.26 (4.86)
	Self-comfort with men’s inappropriate behaviors (normative beliefs)	32.68 (10.82)	33.55 (10.71)
	Rape myth acceptance	35.88 (10.43)	36.69 (10.29)
	Outcome expectancies for engaging in rape	58.71 (10.76)	58.02 (11.17)
	Empathy for rape victims	68.96 (9.66)	68.78 (9.79)
	Hostility toward women^a^	3.42 (2.43)	3.89 (2.54)
	Date rape attitudes	128.85 (24.07)	128.45 (25.84)
	Hyper-gender ideology	46.59 (11.28)	46.38 (11.30)

^a^ Denotes differences in baseline responses between conditions (*P*<.15).

#### Differences Between Completers and Noncompleters

An analysis of sample attrition was conducted to better understand differences between participants who completed the 6-month follow-up and those who did not. A total of 528 participants were lost to follow-up (71.1%), 275 of 367 (74.9%) in the comparison condition versus 253 of 376 (67.3%) in the RealConsent condition (*P*=.02). Comparisons of sociodemographic variables and primary outcomes on baseline responses indicated that completers were more likely to have higher grade point averages (GPAs) than noncompleters (*P*=.01). Completers and noncompleters did not differ on primary outcomes. Inference of GEE model results carries with it an assumption that missing data are missing completely at random (MCAR; more conservative assumption that “missingness” is independent of observed and missing outcomes), whereas our data were missing at random (MAR; less conservative assumption that missingness is only independent of observed outcomes). To investigate whether inference changed as a result of attrition (MAR vs MCAR), missing outcomes were imputed and no change in inference was found; thus, results using original data are presented.

### Primary Outcomes

**Figure 3:**
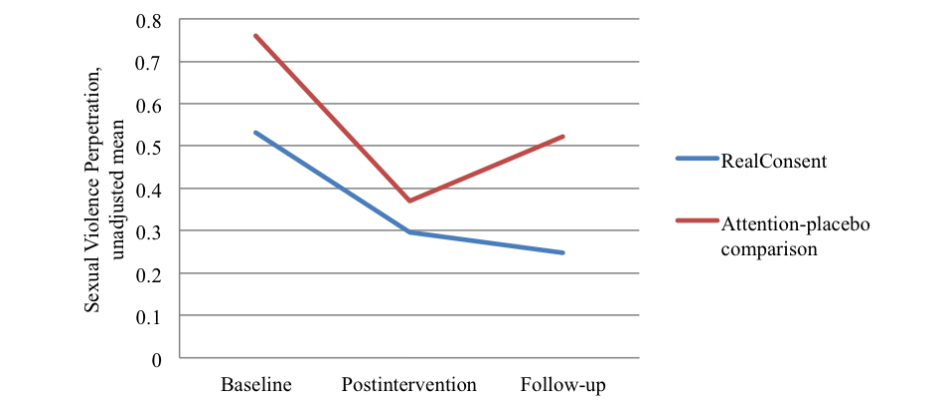
Unadjusted means for sexual violence perpetration across 3 time points for RealConsent and attention-placebo comparison conditions.

**Figure 4:**
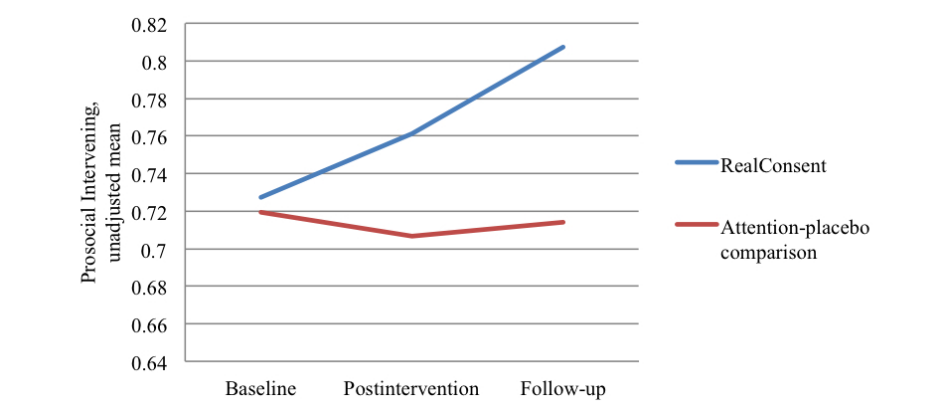
Unadjusted means for prosocial intervening behavior across 3 time points for RealConsent and attention-placebo comparison conditions.

The effectiveness of RealConsent on prosocial intervening behaviors and sexual coercion was estimated with ANCOVA; covariates included baseline scores for the behaviors and those correlated sociodemographic variables. Unadjusted means were graphed across all 3-survey time points and are presented in Figures 3 and 4. RealConsent participants (adjusted mean 0.81, SE 0.03) reported significantly more prosocial intervening behaviors at 6-month follow-up than comparison participants (adjusted mean 0.72, SE 0.03; *F*_1,123_=4.128, *P*=.04). Additionally, RealConsent participants reported significantly less sexual violence at 6-month follow-up (adjusted mean 0.26, SE 0.08) than comparison participants (adjusted mean 0.50, SE 0.09; *F*_1,193_=4.18, *P*=.04). Cohen’s *d* effect sizes for prosocial intervening behaviors and sexual coercion were 0.37 and 0.29, respectively.

Using logistic regression, we assessed the primary prevention effect (ie, comparing those who had not perpetrated to those who had) of RealConsent on prevalence of perpetrating sexual violence. The odds for perpetrating among RealConsent participants were 73% lower than participants in the comparison condition (AOR 0.27, 95% CI 0.11-0.70, *P*=.007).

### Secondary Outcomes/Mediators

Separate GEE analyses were conducted to examine the effect of RealConsent on the 12 proposed mediators. Similar to the previous analyses, we controlled for baseline scores for each mediator and sociodemographic variables and compared the adjusted means of each group over the entire 6-month follow-up period. Of the 12 mediators, only self-efficacy to intervene was not significant (see Table 3).

**Table 3:** Generalized estimating equation (GEE) regression models of intervention effects on psychosocial mediators.

Psychosocial mediators	Estimate (SE)	*P*	RealConsent, adjusted mean (95% CI)^a^	Comparison, adjusted mean (95% CI)^a^
Legal knowledge of assault/rape	1.99 (0.18)	<.001	6.64 (6.41, 6.86)	4.65 (4.38, 4.92)
Knowledge effective consent for sex	0.80 (0.19)	<.001	12.83 (12.59, 13.07)	12.02 (11.74, 12.31)
Self-efficacy to intervene	1.45 (1.57)	.36	91.01 (89.07, 92.95)	89.56 (87.21, 91.92)
Intentions to intervene	1.87 (0.87)	.04	54.72 (53.61, 55.82)	52.84 (51.52, 54.17)
Outcome expectancies for intervening	1.25 (0.38)	.001	29.08 (28.61, 29.55)	27.83 (27.26, 28.40)
Self-comfort with men’s inappropriate behaviors (normative beliefs)	–2.85 (0.83)	.001	30.87 (29.83,31.91)	33.72 (32.49, 34.95)
Rape myth acceptance	–5.48 (0.77)	<.001	31.14 (30.18, 32.10)	36.62 (35.48, 37.76)
Outcome expectancies for rape	1.25 (0.58)	.03	55.12 (54.42, 55.82)	53.86 (52.98, 54.74)
Empathy for rape victims	3.51 (0.73)	<.001	72.04 (71.14, 72.93)	68.52 (67.43, 69.62)
Hostility toward women	–.46 (0.18)	.01	2.74 (2.51, 2.97)	3.20 (2.93, 3.47)
Date rape attitudes	–13.67 (2.08)	<.001	112.36 (109.75, 114.97)	126.03 (122.94, 129.12)
Hyper-gender ideology	–3.67 (0.78)	<.001	42.49 (41.53, 43.46)	46.17 (44.99, 47.34)

^a^ All GEE models included the covariates international student status, fraternity membership, frequency of vaginal intercourse, GPA, residence status, and relationship status.

## Discussion

### Principal Findings

This study was the first Web-based, sexual violence prevention program that incorporated a bystander approach and demonstrated significant changes in behavior for an ethnically diverse sample of male college students. Over the course of the 6-month follow-up period, RealConsent participants were significantly less likely to engage in sexual violence perpetration and significantly more likely to engage in prosocial intervening behavior when they encountered a situation in which they could intervene. It was also observed that these primary behavioral outcomes might have been achieved through hypothesized effects on a host of the program’s theoretical mediators. We found significant changes in all but 1 of the mediating variables and all in the hypothesized direction. This success is noteworthy given that many previous evaluations of sexual violence prevention programs for male college students have measured mostly behavioral intentions rather than actual behavior as outcomes [65,66]; even when behaviors were measured, rarely were significant effects observed [13]. Further, the online administration of the RealConsent program, during which no face-to-face interaction with study participants occurred, suggests that RealConsent’s mode of delivery is equally effective as other approaches involving face-to-face interaction. Thus, given a Web-based modality, RealConsent provides significant advantages by facilitating dissemination thereby increasing reach [22].

Several mechanisms could explain how RealConsent not only increased prosocial intervening behaviors, but also showed effects on sexual violence perpetration. Research has shown theory to be a significant factor in contributing to behavior change among Web-based interventions [67]. RealConsent incorporated social cognitive theory, social norms theory, and the bystander educational model as an overarching framework for developing activities and interactive segments that putatively supported behavior change and identified relevant constructs to be targeted, such as knowledge and self-efficacy, and suggested the correcting of inaccurate perceived norms. Current research documents that misperceptions in norms are a major barrier to bystander intervention and also that perpetrators overestimate other men’s support for what they do [13]. Correcting misperceptions along with teaching bystander intervention combines 2 evidence-based approaches that together may have produced the observed behavioral outcomes. In addition, this framework provided specific behavior change techniques (eg, evoking vicarious learning of targeted behaviors). Because we found significant effects in the hypothesized direction in all but 1 of the theoretical mediators that represented these constructs, it is plausible that several of these theoretical mediating variables would explain the observed behavioral effects. Future research should determine the specific theoretical mediating mechanisms underlying the effects of RealConsent on the primary outcomes.

The efficacy of RealConsent may also be partly attributable to the extensive formative qualitative research conducted by the authors with the targeted population to inform the relevance of content and messages and the style of the Web portal. This formative research, in turn, provided the authors with the necessary resources to build each module of RealConsent. The research team also sought to gain insight from multiple disciplines in the development of the modules; thus, the team included experts from public health, the social sciences, Web design, and marketing. This integration of expertise contributed to the use of proven behavior change techniques that span multiple fields, such as educational entertainment, didactic methods, and problem-based learning with user-face interactivity [68,69] to evoke targeted behavioral outcomes.

Furthermore, because most previous prevention programs have been tested with mostly white participants [65,66], it is important to note that RealConsent showed significant effects among a racially diverse sample of male undergraduates recruited from a large, urban university in the southeast United States. In developing RealConsent, although given no budget constraints, it would have been preferable to reflect deep structure in terms of cultural sensitivity in the content and messages [70]; we were at least able to provide surface structure by using diverse actors and appropriate language in several segments, including the serial drama as well as still images. Appealing to a diverse population provides enhanced generalizability of the program and suggests that RealConsent may be effective with a diverse audience.

These results highlight an important contribution to the literature on evidence-based public health approaches to prevent sexual violence. A review by researchers at the Centers for Disease Control and Prevention (CDC) identified the lack of evidence-based sexual violence prevention approaches at all levels of the social ecology [71]. In particular, they found few effective community-level approaches that seek to alter the characteristics of settings by changing community-level norms. A main tenet of the bystander approach is to provide resources to individuals that may influence their willingness to intervene in situations either actively or passively and to change perceptions of inaccurate norms. Although RealConsent is mostly targeted at changing individual norms and behavior, the effects on intentions to intervene and prosocial intervening behaviors may aggregate to community-level change. Future research could potentially test the effects at the college level to determine whether RealConsent’s effects would reach beyond the male participants enrolled.

### Limitations and Strengths

This study is not without limitations. First, there was a significant amount of attrition mostly because of funding issues and some loss to follow-up. Developing the content, producing the content, and programming the Web portal and the online recruitment platform, in addition to implementing a RCT with a 6-month follow-up took much longer than the anticipated 3-year time frame. Unfortunately, the trial ended prematurely resulting in significant and unforeseen “forced” attrition at 6-month follow-up. Second, we experienced some loss to follow-up. It is unclear what the potential reasons were for this loss to follow-up because data for noncompletion were not collected; however, previous research has shown that attrition in Web-based trials may be higher compared to in-person trials [72,73] and high dropout rates may be considered “a natural and typical feature” [73]. Most important, however, is that our statistical analyses comparing completers and noncompleters on baseline responses indicated 1 minor difference (GPA), which had no influence on study outcomes, and we imputed missing outcomes and no change in inference was found suggesting attrition bias is not a significant threat to the results observed.

Significant differences at baseline on several primary outcomes suggest that randomization was not perfect; however, we have no reason to believe that these differences were not due to chance because participants were assigned by computer algorithm. Nonetheless, we controlled for baseline scores in all analyses and still found significant differences between groups. Lastly, the sample selected for this trial was specific to a large, urban university in the Southeast so results may not generalize to other populations. Future research should replicate these results with populations in both urban and rural universities.

Nevertheless, there were some inherent strengths. The theoretically derived intervention was developed using extensive formative research with the study population and pilot tested with much success and praise. In addition, the RCT coupled with random probability sampling and the use of an attention-placebo comparison group represent significant methodological strengths.

### Conclusions

Given that colleges and universities that receive federal aid are mandated via the Clery Act (20 USCA § 1092) to inform students about crime statistics as well as policies and procedures that are in place to prevent sexual offenses, evidence-based sexual violence prevention programs that are cost-effective, easily implemented, and appeal to a diverse population are much needed. Prior studies have presented numerous sexual violence prevention programs that are effective in changing negative attitudes, rape myths, and behaviors for some; however, none involved an empirically tested Web-based program [20,65,66]. Recent reviews of sexual violence prevention programs describe the importance of engaging men to be women’s allies in preventing sexual violence as an important element, as in a bystander approach [66]; however, equally important is the ability of interventions to reach large populations rather than only the men who volunteer [66]. RealConsent is a scalable intervention that with its Web-based approach, behavioral outcomes, and additional conclusive research holds potential to reach large segments of male undergraduate populations while also engaging young men to intervene so that sexual violence will be prevented.

## Multimedia Appendix 1

CONSORT-EHEALTH checklist V1.6.2 [74].

## Abbreviations

AOR: adjusted odd ratios
CTS2: Revised Conflict Tactics Scale
GEE: generalized estimating equation
GPA: grade point average
MAR: missing at random
MCAR: missing completely at random
RCT: randomized controlled trial
ROLB: Reactions to Offensive Language and Behavior

## References

[1] Black MC, Basile KC, Breiding MJ (2011). The National Intimate Partner and Sexual Violence Survey (NISVS): 2010 Summary report.

[2] Casey EA, Lindhorst TP (2009). Toward a multi-level, ecological approach to the primary prevention of sexual assault: prevention in peer and community contexts. Trauma Violence Abuse.

[3] Amar AF, Gennaro S (2005). Dating violence in college women: associated physical injury, healthcare usage, and mental health symptoms. Nurs Res.

[4] Basile KC, Saltzman LE (2009). Sexual violence surveillance: Uniform definitions and recommended data elements.

[5] Abbey A, McAuslan P (2004). A longitudinal examination of male college students' perpetration of sexual assault. J Consult Clin Psychol.

[6] Fisher BS, Cullen FT, Turner MG (2000). The Sexual Vicitimization of College Women.

[7] Forke CM, Myers RK, Catallozzi M, Schwarz DF (2008). Relationship violence among female and male college undergraduate students. Arch Pediatr Adolesc Med.

[8] Banyard VL (2008). Sexual violence: current perspectives on prevention and intervention. J Prev Interv Community.

[9] Banyard VL, Plante EG, Moynihan MM (2003). Bystander education: Bringing a broader community perspective to sexual violence prevention. J Community Psychol.

[10] Ward KJ (2001). Mentors in Violence Prevention: Evaluation 1999/2000.

[11] Tabachnick J (2009). Engaging bystanders in sexual violence prevention.

[12] Foubert JD, Langhinrichsen-Rohling J, Brasfield H, Hill B (2010). Effects of a rape awareness program on college women: increasing bystander efficacy and willingness to intervene. J Community Psychol.

[13] Gidycz CA, Orchowski LM, Berkowitz AD (2011). Preventing sexual aggression among college men: an evaluation of a social norms and bystander intervention program. Violence Against Women.

[14] Potter SJ (2012). Using a multimedia social marketing campaign to increase active bystanders on the college campus. J Am Coll Health.

[15] Potter SJ, Stapleton JG (2011). Bringing in the target audience in bystander social marketing materials for communities: suggestions for practitioners. Violence Against Women.

[16] Ahrens CE, Rich MD, Ullman JB (2011). Rehearsing for real life: the impact of the InterACT Sexual Assault Prevention Program on self-reported likelihood of engaging in bystander interventions. Violence Against Women.

[17] Langhinrichsen-Rohling J, Foubert JD, Brasfield HM, Hill B, Shelley-Tremblay S (2011). The Men's Program: does it impact college men's self-reported bystander efficacy and willingness to intervene?. Violence Against Women.

[18] Coker AL, Cook-Craig PG, Williams CM, Fisher BS, Clear ER, Garcia LS, Hegge LM (2011). Evaluation of Green Dot: an active bystander intervention to reduce sexual violence on college campuses. Violence Against Women.

[19] Moynihan MM, Banyard VL, Arnold JS, Eckstein RP, Stapleton JG (2011). Sisterhood may be powerful for reducing sexual and intimate partner violence: an evaluation of the Bringing in the Bystander in-person program with sorority members. Violence Against Women.

[20] Banyard VL, Moynihan MM, Crossman M (2009). Reducing sexual violence on campus: the role of student leaders as empowered bystanders. Journal of College Student Development.

[21] Wantland DJ, Portillo CJ, Holzemer WL, Slaughter R, McGhee EM (2004). The effectiveness of Web-based vs. non-Web-based interventions: a meta-analysis of behavioral change outcomes. J Med Internet Res.

[22] Christensen H, Calear AL, Andersson G, Thorndike FP, Tait RJ (2012). Beyond efficacy: the depth and diversity of current internet Interventions. J Med Internet Res.

[23] Cassell MM, Jackson C, Cheuvront B (1998). Health communication on the Internet: an effective channel for health behavior change?. J Health Commun.

[24] Cugelman B, Thelwall M, Dawes P (2011). Online interventions for social marketing health behavior change campaigns: a meta-analysis of psychological architectures and adherence factors. J Med Internet Res.

[25] Evers KE, Prochaska JM, Prochaska JO, Driskell MM, Cummins CO, Velicer WF (2003). Strengths and weaknesses of health behavior change programs on the internet. J Health Psychol.

[26] Muñoz RF (2010). Using evidence-based internet interventions to reduce health disparities worldwide. J Med Internet Res.

[27] Christensen H, Leach LS, Barney L, Mackinnon Aj, Griffiths Km (2006). The effect of web based depression interventions on self reported help seeking: randomised controlled trial [ISRCTN77824516]. BMC Psychiatry.

[28] Kenardy J, McCafferty K, Rosa V (2003). Internet-delivered indicated prevention for anxiety disorders: a randomized controlled trial. Behav Cognit Psychother.

[29] Zabinski MF, Pung MA, Wilfley DE, Eppstein DL, Winzelberg AJ, Celio A, Taylor CB (2001). Reducing risk factors for eating disorders: targeting at-risk women with a computerized psychoeducational program. Int J Eat Disord.

[30] Maes L, Vereecken CA, Gedrich K, Rieken K, Sichert-Hellert W, De Bourdeaudhuij I, Kersting M, Manios Y, Plada M, Hagströmer M, Dietrich S, Matthys C, HELENA Study Group (2008). A feasibility study of using a diet optimization approach in a web-based computer-tailoring intervention for adolescents. Int J Obes (Lond).

[31] Celio AA, Winzelberg AJ, Wilfley DE, Eppstein-Herald D, Springer EA, Dev P, Taylor CB (2000). Reducing risk factors for eating disorders: comparison of an Internet- and a classroom-delivered psychoeducational program. J Consult Clin Psychol.

[32] Riper H, Kramer J, Smit F, Conijn B, Schippers G, Cuijpers P (2008). Web-based self-help for problem drinkers: a pragmatic randomized trial. Addiction.

[33] Kypri K, Saunders JB, Williams SM, McGee RO, Langley JD, Cashell-Smith ML, Gallagher SJ (2004). Web-based screening and brief intervention for hazardous drinking: a double-blind randomized controlled trial. Addiction.

[34] Bewick BM, Trusler K, Mulhern B, Barkham M, Hill AJ (2008). The feasibility and effectiveness of a web-based personalised feedback and social norms alcohol intervention in UK university students: a randomised control trial. Addict Behav.

[35] Postel MG, de Haan HA, ter Huurne ED, Becker ES, de Jong CA (2010). Effectiveness of a web-based intervention for problem drinkers and reasons for dropout: randomized controlled trial. J Med Internet Res.

[36] Riper H, Spek V, Boon B, Conijn B, Kramer J, Martin-Abello K, Smit F (2011). Effectiveness of E-self-help interventions for curbing adult problem drinking: a meta-analysis. J Med Internet Res.

[37] Lenert L, Muñoz RF, Stoddard J, Delucchi K, Bansod A, Skoczen S, Pérez-Stable EJ (2003). Design and pilot evaluation of an internet smoking cessation program. J Am Med Inform Assoc.

[38] Danielson CK, McCauley JL, Jones AM, Borkman AL, Miller S, Ruggiero KJ (2013). Feasibility of delivering evidence-based HIV/STI prevention programming to a community sample of African American teen girls via the internet. AIDS Educ Prev.

[39] Mouthaan J, Sijbrandij M, de Vries GJ, Reitsma JB, van de Schoot R, Goslings JC, Luitse JS, Bakker FC, Gersons BP, Olff M (2013). Internet-based early intervention to prevent posttraumatic stress disorder in injury patients: randomized controlled trial. J Med Internet Res.

[40] Bandura A (2004). Health promotion by social cognitive means. Health Educ Behav.

[41] Fabiano PM, Perkins HW, Berkowitz A, Linkenbach J, Stark C (2003). Engaging men as social justice allies in ending violence against women: evidence for a social norms approach. J Am Coll Health.

[42] Banyard VL, Moynihan MM, Plante EG (2007). Sexual violence prevention through bystander education: An experimental evaluation. J Community Psychol.

[43] The ISA Group.

[44] Streiner DL (2003). Being inconsistent about consistency: when coefficient alpha does and doesn't matter. J Pers Assess.

[45] Hennessy M, Bleakley A, Fishbein M (2012). Measurement Models for Reasoned Action Theory. Ann Am Acad Pol Soc Sci.

[46] Kilmartin C, Conway A, Friedberg A (1999). Social conformity and sexism in all-male peer groups. Virginia Psychological Association Spring Convention.

[47] Loh C, Gidycz CA, Lobo TR, Luthra R (2005). A prospective analysis of sexual assault perpetration: risk factors related to perpetrator characteristics. J Interpers Violence.

[48] Straus MA, Hamby SL, Boney-McCoy S, Sugarman DB (1996). The Revised Conflict Tactics Scales (CTS2): development and preliminary psychometric data. Journal of Family Issues.

[49] Maxwell CD, Robinson AL, Post LA (2003). The nature and predictors of sexual victimization and offending among adolescents. Journal of Youth and Adolescence.

[50] Payne DL, Lonsway KA, Fitzgerald LF (1999). Rape myth acceptance: exploration of its structure and its measurement using the Illinois Rape Myth Acceptance Scale. Journal of Research in Personality.

[51] Hamburger ME, Hogben M, McGowan S, Dawson LJ (1996). Assessing hypergender ideologies: development and initial validation of a gender-neutral measure of adherence to extreme gender-role beliefs. Journal of Research in Personality.

[52] Deitz SR, Blackwell KT, Daley PC, Bentley BJ (1982). Measurement of empathy toward rape victims and rapists. J Pers Soc Psychol.

[53] Check JVP (1985). The Hostility Toward Women Scale [dissertation].

[54] Burgess GH (2007). Assessment of rape-supportive attitudes and beliefs in college men: development, reliability, and validity of the rape attitudes and beliefs scale. J Interpers Violence.

[55] Cohen J (1988). Statistical Power for the Behavioral Sciences. 2nd Ed.

[56] Piantadosi S (1997). Clinical Trials: A Methodologic Perspective.

[57] Pocock SJ (1993). Clinical Trials.

[58] Fleiss JL, Levin B, Paik MC (2003). Statistical Methods for Rates and Proportions. 3rd ed.

[59] Hosmer DW, Lemeshow S (1989). Applied Logisitic Regression.

[60] Kleinbaum DG, Kupper LL, Muller KE, Nizam A (1998). Applied Regression Analysis and Other Multivariable Methods. 3rd ed.

[61] Wilson DB (2013). Practical meta-analysis effect size calculator.

[62] Hardin JW, Hilbe JM (2003). Generalized Estimating Equations.

[63] Liang K, Zeger SL (1986). Longitudinal data analysis using generalized linear models. Biometrika.

[64] Efron B (1981). Nonparametric estimates of standard error: The jackknife, the bootstrap and other methods. Biometrika.

[65] Vladutiu CJ, Martin SL, Macy RJ (2011). College- or university-based sexual assault prevention programs: a review of program outcomes, characteristics, and recommendations. Trauma Violence Abuse.

[66] Garrity SE (2011). Sexual assault prevention programs for college-aged men: a critical evaluation. J Forensic Nurs.

[67] Webb TL, Joseph J, Yardley L, Michie S (2010). Using the internet to promote health behavior change: a systematic review and meta-analysis of the impact of theoretical basis, use of behavior change techniques, and mode of delivery on efficacy. J Med Internet Res.

[68] Abraham C, Michie S (2008). A taxonomy of behavior change techniques used in interventions. Health Psychol.

[69] Bandura A, Bryant J, Zillmann D (1994). Social cognitive theory of mass communication. Media Effects: Advances in Theory and Research.

[70] Resnicow K, Baranowski T, Ahluwalia JS, Braithwaite RL (1999). Cultural sensitivity in public health: defined and demystified. Ethn Dis.

[71] DeGue S, Holt MK, Massetti GM, Matjasko JL, Tharp AT, Valle LA (2012). Looking ahead toward community-level strategies to prevent sexual violence. J Womens Health (Larchmt).

[72] Peels DA, Bolman C, Golsteijn RH, De Vries H, Mudde AN, van Stralen MM, Lechner L (2012). Differences in reach and attrition between Web-based and print-delivered tailored interventions among adults over 50 years of age: clustered randomized trial. J Med Internet Res.

[73] Eysenbach G (2005). The law of attrition. J Med Internet Res.

[74] Eysenbach G, CONSORT-EHEALTH Group (2011). CONSORT-EHEALTH: improving and standardizing evaluation reports of Web-based and mobile health interventions. J Med Internet Res.

