# Supplementary material for: A Web-Based Sexual Violence Bystander Intervention for Male College Students: Randomized Controlled Trial
Source: J Med Internet Res. 2014 Sep 5;16(9):e203. doi: 10.2196/jmir.3426 (PMC4180355; doi:10.2196/jmir.3426)
Supplement: Supplementary file 1 [file jmir_v16i9e203_app1.pdf]

## CONSORT-EHEALTH Checklist V1.6.2 Report

**(based on CONSORT-EHEALTH V1.6), available at [http://tinyurl.com/consort-ehealth-v1-6].**

3426

**Date completed**

7/11/2014 13:36:09

**by**

Laura F. Salazar

A Web-based Sexual Violence Bystander Intervention for Male College Students: A Randomized Controlled Trial

### TITLE

**1a-i) Identify the mode of delivery in the title**

yes

"A Web-based Sexual Violence Bystander Intervention for Male College Students.."

**1a-ii) Non-web-based components or important co-interventions in title**

Not applicable as there are no non-web-based components

**1a-iii) Primary condition or target group in the title**

yes

"A Web-based Sexual Violence Bystander Intervention for Male College Students"

### ABSTRACT

**1b-i) Key features/functionalities/components of the intervention and comparator in the METHODS section of the ABSTRACT**

Yes

"Bystander intervention approaches offer promise for reducing rates of sexual violence (SV) on college campuses. Most interventions are in-person, small group formats limiting their reach and reducing overall public health impact."

"This study evaluated the efficacy of RealConsent, a web-based bystander approach to SV prevention, in enhancing prosocial intervening behaviors and preventing SV perpetration."

**1b-ii) Level of human involvement in the METHODS section of the ABSTRACT**

Yes

"RealConsent was delivered via a password-protected web portal that contained six 30-minute media-based and interactive modules "

**1b-iii) Open vs. closed, web-based (self-assessment) vs. face-to-face assessments in the METHODS section of the ABSTRACT**

Yes

"A random probability sample of male undergraduate students (ages 18 to 24) attending a large, urban university located in the southeastern United States was recruited online and randomized to either: RealConsent (n=376) or a web-based general health promotion program (n=367)"

**1b-iv) RESULTS section in abstract must contain use data**

Yes,

"A random probability sample of male undergraduate students (ages 18 to 24) attending a large, urban university located in the southeastern United States was recruited online and randomized to either: RealConsent (n=376) or a web-based general health promotion program (n=367)"

**1b-v) CONCLUSIONS/DISCUSSION in abstract for negative trials**

Not applicable as positive effects were found.

### INTRODUCTION

**2a-i) Problem and the type of system/solution**

Yes

"To date, there have been no web-based SV prevention programs that target male college students and incorporate the bystander approach that also have been tested with a true experimental design and measure sexual violence as an outcome. In response, RealConsent, a web-based SV prevention program incorporating a bystander approach was developed and tested for its efficacy. "

**2a-ii) Scientific background, rationale: What is known about the (type of) system**

Yes

"To combat the problem of SV, most prevention and intervention programs have therefore focused on college populations and recently have shifted efforts to target elements in the environment rather than solely targeting individual characteristics of perpetrators or victims. The bystander model is one such approach. It is a theoretical model of community-level change, which targets community members to intervene actively in situations that may be harmful and engages them to be accountable and to take action.[9-11] In fact, bystander intervention programs applied to dating and/or sexual violence interventions have proliferated in the past five years.[12-20] Most of these have involved a small-group format (e.g., workshops) whereas a few involved a localized social marketing campaign. Subsequent evaluations demonstrated that the bystander model, in some cases, is effective in promoting active bystander behaviors and in changing social norms.[13]

As promising as these interventions are, due to their small group, in–person format, they are resource-intensive and limited in their reach and sustainability. Alternatively, the use of the Internet as an effective medium to deliver health-related interventions has emerged,[21] and offers significant advantages over in–person interventions such as lower cost of intervention delivery, greater reach, maintenance of fidelity, the possibility of delivery in a wide range of settings, and ability to tailor content to a variety of users.[22-24]"

### METHODS

**3a) CONSORT: Description of trial design (such as parallel, factorial) including allocation ratio**

Yes

"This paper reports primary and secondary outcomes from a randomized controlled trial (RCT) of RealConsent. It was hypothesized that participants randomized to receive the RealConsent program would report greater increases in self-reported prosocial intervening behaviors and fewer incidents of sexual violence perpetration in comparison to participants randomized to receive an attention-placebo comparison program."

**3b) CONSORT: Important changes to methods after trial commencement (such as eligibility criteria), with reasons**

Not Applicable.

There were no changes made to methods after trial commencement.

**3b-i) Bug fixes, Downtimes, Content Changes**

Not applicable

There were no changes made due to bug fixes or other issues.

**4a) CONSORT: Eligibility criteria for participants**

Yes

"Eligible participants were male undergraduates ages 18 to 24, single, who self-reported being either heterosexual or bisexual; exclusion criteria were graduate student status and homosexual sexual orientation"

**4a-i) Computer / Internet literacy**

Not applicable
The population from which the sample was drawn are male college students who have computer/internet literacy.
**4a-ii) Open vs. closed, web-based vs. face-to-face assessments:**
Yes
"Active recruitment began February 2010 and ended in April 2010, and was accomplished through e-mail messages from the principal investigator's university e-mail address sent to randomly selected students. The sampling frame to generate the random sample was a list of student names obtained from the university's Office of Legal Affairs. "

"Email invitations included a link to a website that included a short description of the study that blinded participants to the research questions and a short screener to determine eligibility. Eligible students were directed to a webpage that contained the informed consent form. Students were told that the purpose of the study was to "test multi-media, web-based interactive programs designed for male college students." They were also told that the study would be anonymous (i.e., email addresses would not be linked to their user id or to their responses on the survey). "
**4a-iii) Information giving during recruitment**
Yes,
"Email invitations included a link to a website that included a short description of the study that blinded participants to the research questions and a short screener to determine eligibility. Eligible students were directed to a webpage that contained the informed consent form. Students were told that the purpose of the study was to "test multi-media, web-based interactive programs designed for male college students." They were also told that the study would be anonymous (i.e., email addresses would not be linked to their user id or to their responses on the survey). Participants who provided consent were then directed to the web portal to register and obtain a user name and set their password."
**4b) CONSORT: Settings and locations where the data were collected**
Yes
"Participants who provided consent were then directed to the web portal to register and obtain a user name and set their password. Upon registering, simple randomization was implemented using a customized algorithm that assigned participants to either the experimental condition (RealConsent) or to an attention-placebo comparison condition. Participants were then directed to complete the baseline online survey."
**4b-i) Report if outcomes were (self-)assessed through online questionnaires**
Yes
"Participants were then directed to complete the baseline online survey. Following completion of the survey, they were directed to either the RealConsent program or to the comparison where they could begin the modules immediately or at a later time. Participants were asked to complete a post-intervention and a 6-months post-intervention survey"
**4b-ii) Report how institutional affiliations are displayed**
Not applicable
**5) CONSORT: Describe the interventions for each group with sufficient details to allow replication, including how and when they were actually administered**
**5-i) Mention names, credential, affiliations of the developers, sponsors, and owners**
Yes
"Experimental intervention. The content for RealConsent was based on several complementary theoretical frameworks (social cognitive theory,[35] social norms theory,[36] and the bystander educational model[37]) as well as extensive formative research with the targeted population. RealConsent has two primary goals; (1) to increase prosocial intervening behaviors that reduce risk for SV perpetration (e.g., expressing disapproval when a peer is verbally disparaging toward women; attempting to stop a peer who tries to be coercive/violent) and (2) to prevent sexually violent behaviors toward women. These goals were to be achieved by affecting theoretically and empirically derived mediators such as: increasing knowledge of and skills for safely intervening, correcting misperceptions in normative beliefs, affecting negative attitudes toward date rape, increasing knowledge of the elements constituting informed consent to have sex, affecting masculine gender roles, enhancing communication skills, and increasing empathy for victims of sexual assault."

"Attention-Placebo Comparison intervention. The comparison condition involved a web-based, general health promotion program titled Health Connection, which was developed by the ISA Group (www.isagroup.com). The Health Connection program is an on-line, multi-media, health promotion program with four primary modules -- stress management, fitness, weight management/nutrition and substance abuse. Each program module is approximately 45 minutes long and audio narrated and approximated RealConsent in intensity, format, and time duration."

"Laura Salazar was the developer of RealConsent, but she did not derive financial income from the web-based program. "
**5-ii) Describe the history/development process**
Yes
"The content for RealConsent was based on several complementary theoretical frameworks (social cognitive theory,[35] social norms theory,[36] and the bystander educational model[37]) as well as extensive formative research with the targeted population"
**5-iii) Revisions and updating**
The content of the RealConsent program was "frozen" during the trial and represents a static intervention.
**5-iv) Quality assurance methods**
Yes
"RealConsent was also programmed so that participants could not skip or click-through segments within each module. Participants were allowed to complete the modules at their own pace but were encouraged via email to complete all modules within three weeks."
**5-v) Ensure replicability by publishing the source code, and/or providing screenshots/screen-capture video, and/or providing flowcharts of the algorithms used**
Yes
A screen shot of the web-based program is provided in Figure 1.
**5-vi) Digital preservation**
Yes
"RealConsent was delivered via a password-protected web portal (www.realconsent.org; see Figure 1 for screen shot) and consisted of six, 30-minute modules with each module ranging in number of segments (1-14) and types of activities with diverse actors and appropriate language."
**5-vii) Access**
Yes
"Participants who provided consent were then directed to the web portal to register and obtain a user name and set their password. Upon registering, simple randomization was implemented using a customized algorithm that assigned participants to either the experimental condition (RealConsent) or to an attention-placebo comparison condition. "

"RealConsent was delivered via a password-protected web portal (www.realconsent.org; see Figure 1 for screen shot) and consisted of six, 30-minute modules with each module ranging in number of segments (1-14) and types of activities with diverse actors and appropriate language."

**5-viii) Mode of delivery, features/functionalities/components of the intervention and comparator, and the theoretical framework**
Yes
"RealConsent was delivered via a password-protected web portal (www.realconsent.org; see Figure 1 for screen shot) and consisted of six, 30-minute modules with each module ranging in number of segments (1-14) and types of activities with diverse actors and appropriate language. Each of the modules involved interactivity, didactic activities and episodes of a serial drama, which allowed us to model positive behaviors and illustrate both positive and negative outcome expectations for intervening and for perpetrating violence against women. Behaviors modeled in the serial drama included intervening, communicating with female sex partners, and obtaining informed consent to have sex."
**5-ix) Describe use parameters**
Yes
"RealConsent was also programmed so that participants could not skip or click-through segments within each module. Participants were allowed to complete the modules at their own pace but were encouraged via email to complete all modules within three weeks. Following the completion of each module, participants were immediately assessed and compensated $10 for providing their acceptability and feedback on the module. These procedures ensured that each participant at follow-up had completed each module in its entirety."
**5-x) Clarify the level of human involvement**
Yes
"The portal also allowed participants to send emails and post messages if they needed technical assistance or had questions about their payments. "
**5-xi) Report any prompts/reminders used**
Yes
"RealConsent was also programmed so that participants could not skip or click-through segments within each module. Participants were allowed to complete the modules at their own pace but were encouraged via email to complete all modules within three weeks. "
**5-xii) Describe any co-interventions (incl. training/support)**
Not applicable
The RealConsent program is a stand-alone intervention.
**6a) CONSORT: Completely defined pre-specified primary and secondary outcome measures, including how and when they were assessed**
Yes
"Primary outcome measures included prosocial intervening behaviors, sexual violence perpetration; secondary outcome measures were a host of theoretical mediating variables linked to the intervention activities. "

"Prosocial Intervening Behaviors were assessed with the Reactions to Offensive Language and Behavior (ROLB) index that measures whether or not men confronted inappropriate behaviors of other men.[39, 40] We used the 7-item self-behavior subscale plus an additional 8 items,[9] which directly reflected the content of RealConsent. A series of 15 potential intervening situations were presented and participants were asked to indicate whether they had experienced this situation (yes/no), whether they had "ever" intervened (at baseline) or whether they had intervened "within the past six months" (at 6-month follow-up)."

"Sexual Violence was assessed with the sexual coercion subscale from the Revised Conflict Tactics Scale (CTS2).[41] The CTS2 sexual coercion subscale is a 7-item questionnaire that assesses sexual coercion perpetration where participants are asked to indicate whether they had engaged in a sexually abusive tactic (e.g., "I used force like hitting, holding down or using a weapon to make a woman have sex") within a certain timeframe. We used "ever" at baseline and "within the past 6 months" at the 6-month follow-up."

"The secondary outcome measures included legal knowledge of assault/rape,[42] knowledge of informed consent to have sex, self-efficacy to intervene,[9, 39] intentions to intervene, outcome expectancies for intervening behaviors,[9] normative beliefs regarding sexual violence toward women, [39, 40] rape myths,[43] gender-role ideology,[44] empathy for rape victims,[45] hostility towards women,[46] attitudes toward date rape,[47] and outcome expectancies for engaging in non-consensual sex. Table 1 provides additional information about these secondary measures including the mean and standard deviation, Cronbach's alpha, the number of items, response options, and a sample item."
**6a-i) Online questionnaires: describe if they were validated for online use and apply CHERRIES items to describe how the questionnaires were designed/deployed**
Yes
The measures used in this study had been previously validated with the population, but were not validated specifically for online use. Cronbach's alpha, a measure of reliability for scales, was calculated and all had acceptable reliability. See Table 1.
**6a-ii) Describe whether and how "use" (including intensity of use/dosage) was defined/measured/monitored**
Yes
"RealConsent was also programmed so that participants could not skip or click-through segments within each module. Participants were allowed to complete the modules at their own pace but were encouraged via email to complete all modules within three weeks. Following the completion of each module, participants were immediately assessed and compensated $10 for providing their acceptability and feedback on the module. These procedures ensured that each participant at follow-up had completed each module in its entirety."
**6a-iii) Describe whether, how, and when qualitative feedback from participants was obtained**
Yes
"Following the completion of each module, participants were immediately assessed and compensated $10 for providing their acceptability and feedback on the module. "
**6b) CONSORT: Any changes to trial outcomes after the trial commenced, with reasons**
Yes
No changes to trial outcomes were made after the trial commenced.
**7a) CONSORT: How sample size was determined**
**7a-i) Describe whether and how expected attrition was taken into account when calculating the sample size**
yes
"Sample size calculations for the primary behavioral outcomes were estimated to guarantee that power would be at least .75 for the detection of small effect sizes.[48] Under a two group design, and assuming 10% attrition over the 6-month follow-up period required enrolling at least 340 in each study condition to detect the specified effect size with power of .83."
**7b) CONSORT: When applicable, explanation of any interim analyses and stopping guidelines**
Not applicable
**8a) CONSORT: Method used to generate the random allocation sequence**
yes
"Participants who provided consent were then directed to the web portal to register and obtain a user name and set their password. Upon registering, simple randomization was implemented using a customized algorithm that assigned participants to either the experimental condition (RealConsent) or to an attention-placebo comparison condition"
**8b) CONSORT: Type of randomisation; details of any restriction (such as blocking and block size)**

Yes
"Upon registering, simple randomization was implemented using a customized algorithm that assigned participants to either the experimental condition (RealConsent) or to an attention-placebo comparison condition"
**9) CONSORT: Mechanism used to implement the random allocation sequence (such as sequentially numbered containers), describing any steps taken to conceal the sequence until interventions were assigned**
Yes
" Upon registering, simple randomization was implemented using a customized algorithm that assigned participants to either the experimental condition (RealConsent) or to an attention-placebo comparison condition"
**10) CONSORT: Who generated the random allocation sequence, who enrolled participants, and who assigned participants to interventions**
Yes
"Participants who provided consent were then directed to the web portal to register and obtain a user name and set their password. Upon registering, simple randomization was implemented using a customized algorithm that assigned participants to either the experimental condition (RealConsent) or to an attention-placebo comparison condition."
**11a) CONSORT: Blinding - If done, who was blinded after assignment to interventions (for example, participants, care providers, those assessing outcomes) and how**
**11a-i) Specify who was blinded, and who wasn't**
Yes
"Email invitations included a link to a website that included a short description of the study that blinded participants to the research questions and a short screener to determine eligibility. Eligible students were directed to a webpage that contained the informed consent form. Students were told that the purpose of the study was to "test multi-media, web-based interactive programs designed for male college students." "
**11a-ii) Discuss e.g., whether participants knew which intervention was the "intervention of interest" and which one was the "comparator"**
Yes
"Email invitations included a link to a website that included a short description of the study that blinded participants to the research questions and a short screener to determine eligibility. Eligible students were directed to a webpage that contained the informed consent form. Students were told that the purpose of the study was to "test multi-media, web-based interactive programs designed for male college students." "
**11b) CONSORT: If relevant, description of the similarity of interventions**
Yes
"RealConsent was delivered via a password-protected web portal (www.realconsent.org; see Figure 1 for screen shot) and consisted of six, 30-minute modules with each module ranging in number of segments (1-14) and types of activities with diverse actors and appropriate language. Each of the modules involved interactivity, didactic activities and episodes of a serial drama"

"The comparison condition involved a web-based, general health promotion program titled Health Connection, which was developed by the ISA Group (www.isagroup.com). The Health Connection program is an on-line, multi-media, health promotion program with four primary modules -- stress management, fitness, weight management/nutrition and substance abuse. Each program module is approximately 45 minutes long and audio narrated and approximated RealConsent in intensity, format, and time duration."
**12a) CONSORT: Statistical methods used to compare groups for primary and secondary outcomes**
Yes
"At baseline, descriptive statistics were calculated to summarize sociodemographic variables, theoretical mediators, intervening and sexual perpetration behaviors between study conditions. Differences between study conditions were assessed using t-tests for continuous variables and $\chi 2$ analyses for categorical variables.[51] Variables in which differences between study conditions approached statistical significance (P <. 10) or that were theoretically or empirically identified as potential confounders were included as covariates in the models. "
**12a-i) Imputation techniques to deal with attrition / missing values**
Yes
"An analysis of sample attrition was conducted to better understand differences between participants who completed the 6-month follow-up and those who did not. A total of 528 participants were lost to follow-up; 275 (75%) in the control condition versus 253 (67%) in the RealConsent condition (P=. 022). Comparisons of sociodemographic variables and primary outcomes on baseline responses indicated that completers were more likely to have higher GPAs than non-completers (P=0.011). Completers and non-completers did not differ on primary outcomes. Inference of GEE model results carries with it an assumption that missing data are missing completely at random (MCAR; more conservative assumption that missingness is independent of observed and missing outcomes), while our data are missing at random (MAR; less conservative assumption that missingness is only indepdent of observed outcomes). To investigate whether inference changed as a result of attrition (MAR versus MCAR), missing outcomes were imputed and no change in inference was found; thus, results using original (non-imputed) data are presented."
**12b) CONSORT: Methods for additional analyses, such as subgroup analyses and adjusted analyses**
Yes
"Variables in which differences between study conditions approached statistical significance (P <. 10) or that were theoretically or empirically identified as potential confounders were included as covariates in the models."

## RESULTS

**13a) CONSORT: For each group, the numbers of participants who were randomly assigned, received intended treatment, and were analysed for the primary outcome**
Yes
This information is provided in the CONSORT flow diagram Figure 2.
**13b) CONSORT: For each group, losses and exclusions after randomisation, together with reasons**
Yes
"The recruitment process resulted in N=1,406 male college students who were screened. Of those, N=295 (21%) were not eligible and N=1,111 (79%) accepted and consented. There was some attrition (33%) between initially agreeing and subsequently completing the baseline survey. The final sample resulted in N=743 eligible students who were randomized and completed baseline. At post-intervention, N=451 (61%) completed the follow-up survey. Attrition at this time point was loss to follow-up. At 6-month follow-up, N=215 (29%) completed the survey and were included in analyses (Figure 2). Attrition at this final time point was due to loss to follow-up, but also because the trial was ended prematurely."
**13b-i) Attrition diagram**
No
We did not provide an attrition diagram for this study.
**14a) CONSORT: Dates defining the periods of recruitment and follow-up**
Yes
"Active recruitment began February 2010 and ended in April 2010,"
"Upon registering, simple randomization was implemented using a customized algorithm that assigned participants to either the experimental condition (RealConsent) or to an attention-placebo comparison condition. Participants were then directed to complete the baseline online survey. Following completion of the survey, they were directed to either the RealConsent program or to the comparison where they could begin the modules immediately or at a later time. Participants were asked to complete a post-intervention and a 6-months post-intervention survey. "

**14a-i) Indicate if critical “secular events” fell into the study period**
Not applicable
To the best of our knowledge, as our sample represented male college students matriculated at one university, this would not be applicable.
**14b) CONSORT: Why the trial ended or was stopped (early)**
yes
"Attrition at this final time point was due to loss to follow-up, but also because the trial was ended prematurely. More information is provided in the Discussion section."
"there was a significant amount of attrition mostly due to funding issues and some loss to follow-up. Developing the content, producing the content, programming the web portal and online recruitment platform, in addition to implementing a RCT with a 6-month follow-up took much longer than the three-year time frame anticipated. Unfortunately, this caused the study team to prematurely end the trial resulting in significant and unforeseen “forced” attrition at 6-month follow-up"
**15) CONSORT: A table showing baseline demographic and clinical characteristics for each group**
Yes
Table 2
**15-i) Report demographics associated with digital divide issues**
Not applicable
The study sample was somewhat homogenous in terms of computer/internet literacy as it was drawn from a population of male undergraduates matriculated at a large, university.
**16a) CONSORT: For each group, number of participants (denominator) included in each analysis and whether the analysis was by original assigned groups**
**16-i) Report multiple “denominators” and provide definitions**
Yes
Number of participants inlcuded in each analysis is provided in Figure 2.
For primary and secondary outcomes, Cohen's D is reported for mean differences and AOR's are reported for binary outcomes.
**16-ii) Primary analysis should be intent-to-treat**
Yes
"Analyses were performed only on pre-specified hypotheses using an intention-to-treat protocol in which participants were analyzed according to their original assigned study conditions."
**17a) CONSORT: For each primary and secondary outcome, results for each group, and the estimated effect size and its precision (such as 95% confidence interval)**
Yes
"The effectiveness of RealConsent on prosocial intervening behaviors and sexual coercion was estimated with ANCOVA; covariates included baseline scores for the behaviors and those correlated sociodemographic variables. Unadjusted means were graphed across all three-survey time points and are presented in Figures 3 and 4. RealConsent participants (Madj= 81.1) reported significantly more prosocial intervening behaviors at 6-month follow-up than control participants (Madj = 72.2) (F1, 123 = 4.128, P =.044). Additionally, RealConsent participants reported significantly less sexual violence at 6-month follow-up (Madj=. 258) than control participants (Madj =. 498) (F1, 193=4.182, P =. 042). Cohen’s d effect sizes for prosocial intervening behaviors and sexual coercion were .37 and .29, respectively."

"Using logistic regression we assessed the primary prevention effect (i.e., comparing those who had not perpetrated to those who had) of RealConsent on prevalence of perpetrating sexual violence. The odds for perpetrating among RealConsent participants were 73% lower than participants in the control condition (AOR=0.273, 95% CI=. 11-.70, P =. 007)."
**17a-i) Presentation of process outcomes such as metrics of use and intensity of use**
yes
"RealConsent was also programmed so that participants could not skip or click-through segments within each module. Participants were allowed to complete the modules at their own pace but were encouraged via email to complete all modules within three weeks. Following the completion of each module, participants were immediately assessed and compensated $10 for providing their acceptability and feedback on the module."
**17b) CONSORT: For binary outcomes, presentation of both absolute and relative effect sizes is recommended**
No
Only the adjusted odds ratios (absolute risk) are provided for primary outcomes that are binary.
**18) CONSORT: Results of any other analyses performed, including subgroup analyses and adjusted analyses, distinguishing pre-specified from exploratory**
Yes
"The effectiveness of RealConsent on prosocial intervening behaviors and sexual coercion was estimated with ANCOVA; covariates included baseline scores for the behaviors and those correlated sociodemographic variables"..."RealConsent participants (Madj= 81.1) reported significantly more prosocial intervening behaviors at 6-month follow-up than control participants (Madj = 72.2) (F1, 123 = 4.128, P =.044). Additionally, RealConsent participants reported significantly less sexual violence at 6-month follow-up (Madj=. 258) than control participants (Madj =. 498) (F1, 193=4.182, P =. 042). Cohen’s d effect sizes for prosocial intervening behaviors and sexual coercion were .37 and .29, respectively."

"Using logistic regression we assessed the primary prevention effect (i.e., comparing those who had not perpetrated to those who had) of RealConsent on prevalence of perpetrating sexual violence. The odds for perpetrating among RealConsent participants were 73% lower than participants in the control condition (AOR=0.273, 95% CI=. 11-.70, P =. 007)."

"Separate GEE analyses were conducted to examine the effect of RealConsent on the 12 proposed mediators. Similar to analyses above, we controlled for baseline scores for each mediator and sociodemographic variables and compared the adjusted means of each group over the entire 6-month follow-up period"
**18-i) Subgroup analysis of comparing only users**
yes
"An analysis of sample attrition was conducted to better understand differences between participants who completed the 6-month follow-up and those who did not. A total of 528 participants were lost to follow-up; 275 (75%) in the control condition versus 253 (67%) in the RealConsent condition (P=. 022). Comparisons of sociodemographic variables and primary outcomes on baseline responses indicated that completers were more likely to have higher GPAs than non-completers (P=0.011). Completers and non-completers did not differ on primary outcomes. Inference of GEE model results carries with it an assumption that missing data are missing completely at random (MCAR; more conservative assumption that missingness is independent of observed and missing outcomes), while our data are missing at random (MAR; less conservative assumption that missingness is only indepdent of observed outcomes). To investigate whether inference changed as a result of attrition (MAR versus MCAR), missing outcomes were imputed and no change in inference was found; thus, results using original (non-imputed) data are presented."
**19) CONSORT: All important harms or unintended effects in each group**

Yes
No adverse events were reported during the course of this trial.
**19-i) Include privacy breaches, technical problems**
Not applicable
We did not experience any privacy breaches or technical problems.
**19-ii) Include qualitative feedback from participants or observations from staff/researchers**
No
We collected qualitative feedback from participants but cannot include these data in this paper due to word count limitations.

## DISCUSSION

**20) CONSORT: Trial limitations, addressing sources of potential bias, imprecision, multiplicity of analyses**
**20-i) Typical limitations in ehealth trials**
Yes
"This study is not without limitations. First and foremost, there was a significant amount of attrition mostly due to funding issues and some loss to follow-up. Developing the content, producing the content, programming the web portal and online recruitment platform, in addition to implementing a RCT with a 6-month follow-up took much longer than the three-year time frame anticipated. Unfortunately, this caused the study team to prematurely end the trial resulting in significant and unforeseen “forced” attrition at 6-month follow-up. Second, we experienced some loss to follow-up. It is unclear what the potential reasons were for this loss to follow-up because data for non-completion were not collected; however, previous research has shown that in general, attrition in web-based trials may be higher compared to in person trials, [71, 72] and high drop-out rates may be considered “a natural and typical feature.” [72] Most important, however, is that our statistical analyses comparing completers and non-completers on baseline responses indicated one minor difference (GPA), which had no influence on study outcomes, and we imputed missing outcomes and no change in inference was found suggesting attrition bias is not a significant threat to the results observed.
Significant differences at baseline on several primary outcomes suggest that randomization was not perfect; however, we have no reason to believe that these differences were not due to chance as participants were assigned by computer algorithm. Nonetheless, we controlled for baseline scores in all analyses and still found significant differences between groups."
**21) CONSORT: Generalisability (external validity, applicability) of the trial findings**
**21-i) Generalizability to other populations**
Yes
"Lastly, the sample selected for this trial was specific to one large, urban university in the Southeast so results may not generalize to other populations. Future research should replicate these results with populations in both urban and rural universities."
**21-ii) Discuss if there were elements in the RCT that would be different in a routine application setting**
No
Participants in this RCT were recruited and then paid for their time and effort completing the programs but also for the online assessments. In a routine application, the lengthy online surveys would not be part of the application nor would incentives.
**22) CONSORT: Interpretation consistent with results, balancing benefits and harms, and considering other relevant evidence**
**22-i) Restate study questions and summarize the answers suggested by the data, starting with primary outcomes and process outcomes (use)**
Yes
"This study was the first web-based, sexual violence prevention program that incorporated a bystander approach and demonstrated significant changes in behavior for an ethnically diverse sample of male college students. Over the course of the 6-month follow-up period, RealConsent participants were significantly less likely to engage in sexual violence perpetration and significantly more likely to engage in prosocial intervening behavior when they encountered a situation in which they could intervene. It was also observed that these primary behavioral outcomes might have been achieved through hypothesized effects on a host of the program’s theoretical mediators. We found significant changes in all but one of the mediating variables and all in the hypothesized direction."
**22-ii) Highlight unanswered new questions, suggest future research**
Yes
"Several mechanisms could explain how RealConsent not only increased prosocial intervening behaviors, but also showed effects on sexual violence perpetration. Research has shown theory to be a significant factor in contributing to behavior change among web-based interventions.[66] RealConsent incorporated social cognitive theory, social norms theory, and the bystander educational model as an overarching framework for developing activities and interactive segments that putatively supported behavior change and identified relevant constructs to be targeted such as knowledge and self-efficacy, and suggested the correcting of inaccurate perceived norms. Current research documents that misperceptions in norms are a major barrier to bystander intervention and also that perpetrators over-perceive other men’s support for what they do.[13] Correcting misperceptions along with teaching bystander intervention combines two evidence-based approaches that together may have produced the observed behavioral outcomes. In addition, this framework provided specific behavior change techniques (e.g., evoking vicarious learning of targeted behaviors). Because we found significant effects in the hypothesized direction in all but one of the theoretical mediators that represented these constructs, it is plausible that several of these theoretical mediating variables would explain the observed behavioral effects.Future research should determine the specific theoretical mediating mechanisms underlying the effects of RealConsent on the primary outcomes."

"While RealConsent is mostly targeted at changing individual norms and behavior, the effects on intentions to intervene and prosocial intervening behaviors may aggregate to community-level change. Future research could potentially test the effects at the college level to determine whether RealConsent reached a higher level beyond the male participants enrolled"

## Other information

**23) CONSORT:  Registration number and name of trial registry**
Yes
"This study is registered at www.clinicaltrials.gov as “A Web-based Bystander Education Program (RealConsent)”, NCT01903876, on July 2013"
**24) CONSORT: Where the full trial protocol can be accessed, if available**
Yes
The trial protocol is accessible on the clinicaltrials.gov website.
**25) CONSORT: Sources of funding and other support (such as supply of drugs), role of funders**
yes
"This study was funded by the National Center for Injury Prevention and Control, Centers for Disease Control and Prevention, Grant # R49 CE000892."
**X26-i) Comment on ethics committee approval**
Yes
"An RCT was implemented at a large, urban university located in Atlanta, GA. Study procedures were approved by participating sites’ Institutional Review Boards. Participants provided informed consent; however, documentation was waived due to online recruitment. "
**x26-ii) Outline informed consent procedures**

Yes
"Email invitations included a link to a website that included a short description of the study that blinded participants to the research questions and a short screener to determine eligibility. Eligible students were directed to a webpage that contained the informed consent form. Students were told that the purpose of the study was to “test multi-media, web-based interactive programs designed for male college students.” They were also told that the study would be anonymous (i.e., email addresses would not be linked to their user id or to their responses on the survey). Participants who provided consent were then directed to the web portal to register and obtain a user name and set their password."

**X26-iii) Safety and security procedures**

Yes
"They were also told that the study would be anonymous (i.e., email addresses would not be linked to their user id or to their responses on the survey)"
"The portal also allowed participants to send emails and post messages if they needed technical assistance or had questions about their payments. "

Also, participants could download a pdf of the informed consent form, which contained the PI's contact information.

**X27-i) State the relation of the study team towards the system being evaluated**

Yes
"Laura Salazar was the developer of RealConsent, but she did not derive financial income from the web-based program. "
